# A benchmark GaoFen-7 dataset for building extraction from satellite images

**DOI:** 10.1038/s41597-024-03009-5

**Published:** 2024-02-10

**Authors:** Peimin Chen, Huabing Huang, Feng Ye, Jinying Liu, Weijia Li, Jie Wang, Zixuan Wang, Chong Liu, Ning Zhang

**Affiliations:** 1 School of Geospatial Engineering and Science, Sun Yat-Sen University, and Southern Marine Science and Engineering Guangdong Laboratory (Zhuhai), Zhuhai, 519082 China; 2 International Research Center of Big Data for Sustainable Development Goals, Beijing, China; 3 Peng Cheng Laboratory, Shenzhen, 518066 China; 4 The Key Laboratory of Natural Resources Monitoring in Tropical and Subtropical Area of South China, Ministry of Natural Resources, Guangdong, 519082 China; 5 Remote Sensing Application Center, Ministry of Housing and Urban-Rural Development of the People’s Republic of China, and China Academy of Urban Planning and Design, Beijing, 100835 China

**Keywords:** Urban ecology, Geography

## Abstract

Accurate building extraction is crucial for urban understanding, but it often requires a substantial number of building samples. While some building datasets are available for model training, there remains a lack of high-quality building datasets covering urban and rural areas in China. To fill this gap, this study creates a high-resolution GaoFen-7 (GF-7) Building dataset utilizing the Chinese GF-7 imagery from six Chinese cities. The dataset comprises 5,175 pairs of 512 × 512 image tiles, covering 573.17 km^2^. It contains 170,015 buildings, with 84.8% of the buildings in urban areas and 15.2% in rural areas. The usability of the GF-7 Building dataset has been proved with seven convolutional neural networks, all achieving an overall accuracy (OA) exceeding 93%. Experiments have shown that the GF-7 building dataset can be used for building extraction in urban and rural scenarios. The proposed dataset boasts high quality and high diversity. It supplements existing building datasets and will contribute to promoting new algorithms for building extraction, as well as facilitating intelligent building interpretation in China.

## Background & Summary

Automatic building extraction from remote sensing imagery has been developed for many years and is important for urban planning, land-use management, urban environment and thermal comfort research^1,2^. The improvements of sensor capabilities, advanced technologies, and computational resources have significantly promoted building extraction research. Traditional building extraction methods, such as pixel-based (e.g., clustering and thresholding) or object-based image classification methods, often encounter difficulties when dealing with practical scenes due to complex and diverse building environments^3^. Deep learning (DL), especially the convolutional neural networks (CNNs) and transformer-based methods, has demonstrated impressive performance in building segmentation. It is proven highly effective in dealing with complicated scenarios as it can distill high-level semantic knowledge from the training data^4–6^. Despite their effectiveness, DL-based building extraction methods heavily rely on sufficient training data that contains prior knowledge of the buildings, especially for real-world applications. Recently, the emergence of large artificial intelligence (AI) models, such as the Segment Anything Model (SAM), has unlocked novel possibilities and avenues for intelligent image understanding, especially for image segmentation tasks^7^. These models are trained on a massive dataset and show a great ability to generate a valid segmentation result for any prompt. However, their potential may be limited when faced with lower spatial resolution satellite images, leading to incomplete and incorrect segmentation^8,9^. In this context, constructing satellite-based building segmentation datasets is of great research significance for promoting real-world building extraction in the remote sensing field.

Many datasets have been developed for building extraction studies, laying the research foundation for intelligent interpretation of buildings in the big data era. In the early years, the majority of building segmentation studies focused on very high resolution (VHR) aerial optical imagery and a series of datasets have been formed, for instance, the Zeebrugge dataset^10^, the AIRS dataset^11^, the Inria aerial image labeling dataset^12^, the Massachusetts building dataset^13^, the ISPRS Potsdam and Vaihingen dataset^14^, and the WHU building dataset-Aerial imagery dataset^15^. These aerial optical datasets boast a very high spatial resolution, but they contain a limited variety of buildings, thereby restricting their applicability in real-world scenarios (Table 1).

**Table 1:** Information of the open-access building segmentation datasets.

Dataset		Resolution (m)	Sample size (pixels)	Sample count	Area (km^2^)	Bands	Vertical feature	Image type	Geographical region
Zeebrugge^10^		0.05	10,000 × 10,000	7	12	RGB	LiDAR	Aerial	Zeebrugge (Belgium)
AIRS^11^		0.075	10,000 × 10,000	1,047	475	RGB	No	Aerial	Christchurch (New Zealand)
Inria Aerial Image Labeling Dataset^12^		0.3	5,000 × 5,000	180	810	RGB	No	Aerial	Cities in the United States and Austria, etc.
Massachusetts^13^		1	1,500 × 1,500	151	340	RGB	No	Aerial	Massachusetts (the United States)
ISPRS^14^	Potsdam	0.05	6,000 × 6,000	38	2	RGB-NIR	DSM	Aerial	Potsdam (Germany)
	Vaihingen	0.09	>1,200 × 1,800	33	11	RGB-NIR	DSM	Aerial	Vaihingen (Germany)
WHU building dataset^15^	Aerial imagery	0.3	512 × 512	8,188	450	RGB	No	Aerial	Christchurch (New Zealand)
	Satellite dataset I (WHU-I) and Satellite dataset II(WHU-II)	0.3–2.5	512 × 512	17,592	950	RGB-NIR	No	Satellite	Global cities (Los Angeles, Ottawa, Cairo, Milan, etc.) and East Asia
SpaceNet 1^16^	Rio de Janeiro	0.5	650 × 650	1,400	2,544	8-band multispectral of WV2	No	Satellite	Rio de Janeiro (Brazil)
SpaceNet 2^16^	Vegas-Paris-Shanghai-Khartoum	0.3	650 × 650	10,863	7,110	8-band multispectral of WV3	No	Satellite	Vegas, Paris, Shanghai, Khartoum
SpaceNet 4^17^		0.3	900 × 900	62,000	665	8-band multispectral of WV3	Multi-view	Satellite	Atlanta (the United States)
Chinese building instance segmentation dataset (CBISD)^19^		0.29	500 × 500	7,260	124.3	RGB	No	Satellite	Beijing, Shanghai, Shenzhen, and Wuhan (China)

Satellite optical building segmentation datasets have become increasingly popular in recent years, driven by the easier accessibility of high-resolution satellite optical imagery, the improved resolution of satellite imagery, and its broader geographical coverage. To date, several satellite optical building segmentation datasets, such as the SpaceNet 1 and 2^16^, the SpaceNet 4^17^, and the WHU building dataset-Satellite dataset I (WHU-I) and Satellite dataset II (WHU-II)^15^, have been freely available. SpaceNet 1 and 2, and SpaceNet 4 encompass wide areas featuring high spatial resolution and diverse spectral bands. WHU-I is a small dataset with extremely diverse building styles and image sources (with resolutions varying from 0.3 m to 2.5 m). In comparison, WHU-II is relatively larger, covering 860 km^2^ with a spatial resolution of 0.45 m, but it only contains a single building type. These datasets enable models to learn robust features better and significantly facilitate building extraction research. However, they are mainly distributed in the Americas, Europe, and East Asia, lacking distribution within China. Furthermore, concerning the label quality, the labels of SpaceNet 1 and 2^16^, and SpaceNet 4^17^ are constructed using OpenStreetMap (OSM), resulting in relatively low annotation accuracies, which impacts the correctness and fairness of evaluating a building extraction model^18^.

To promote further research on building extraction in China, high-quality and large-scale building segmentation datasets are required to be investigated. However, to the best of our knowledge, there are only a few publicly available satellite optical building segmentation datasets for Chinese cities. Fang *et al*.^19^ constructed a Chinese building instance segmentation dataset (CBISD) from Google Earth images. CBISD comprises 7,260 pairs of 500 × 500 images, covering an area of 124.32 km^2^, and containing 63,886 buildings in four Chinese cities (i.e., Beijing, Shanghai, Shenzhen, and Wuhan). While CBISD boasts a high spatial resolution of 0.29 m and is of high quality, it primarily covers urban and suburban buildings, with a small proportion of rural buildings. Huang *et al*.^20^ presented a novel Urban Building Classification (UBC) dataset for building detection and fine-grained classification from the Chinese high-resolution SuperView (or “GaoJing” in Chinese) satellite images and GF-2 images in urban areas of Beijing and Munich. The dataset covers an area of 65.6 km^2^ and contains 41,586 building instances. Additionally, there are some large-scale satellite land-cover datasets available for building extraction, such as the Gaofen Image Dataset (GID) and the Land-cOVEr Domain Adaptive semantic segmentation (LoveDA) dataset^21,22^. These datasets are designed to encompass various types of features, and their annotation for specific features (e.g., buildings) is relatively coarse, thereby constraining their applicability to accurate building extraction research. These aforementioned datasets have prepared the field to address building extraction from satellite images in China to some extent. Nevertheless, there remains a lack of diverse, high-quality, and multi-scene building segmentation datasets covering different regions of China, especially rural areas of China, where some special buildings (of different sizes, shapes, and materials) exist.

Given these issues and challenges, this study introduces the Chinese GaoFen-7 (GF-7) satellite imagery to construct a high-resolution (0.65 m) building segmentation dataset. The Chinese GF-7 satellite image is advantageous in capturing the clear geometrical structure and fine texture of different kinds of buildings, owing to the sub-meter imaging characteristics of the GF-7 satellite. It contains rich spectral information of buildings with red, green, blue, and near-infrared bands. Additionally, the GF-7 image provides extensive coverage, encompassing nearly all regions throughout China. Therefore, it is an optimal data source for constructing building segmentation datasets in China. Our main contributions are summarized as follows:

1. We present a high-resolution (0.65 m) and high-quality building segmentation dataset (i.e., GF-7 Building dataset) using the GF-7 images from six typical Chinese cities.
2. We provide a series of benchmark experimental results for the GF-7 Building dataset and analyze its transferability and limitations.
3. We discuss the potential application of the GF-7 Building dataset for the intelligent interpretation of buildings.

## Methods

### Data collection and pre-processing

The GF-7 satellite images used in the study were distributed by the Land Satellite Remote Sensing Application Center of China (http://www.lasac.cn/). These images were acquired between 2020 and 2022 in Tianjin, Lanzhou, Chongqing, Ningbo, Guangzhou, and Shenzhen, with a cloud cover of fewer than 5%. Each GF-7 image scene contains three images, i.e., one backward multispectral image (blue, green, red, and near-infrared bands) and two panchromatic images (with +26° forward and −5° backward viewing angles). The spatial resolution of the GF-7 backward multispectral imagery is 2.6 m, while the spatial resolution of the GF-7 backward panchromatic imagery is 0.65 m (Table 2). The GF-7 backward panchromatic imagery and backward multispectral imagery were utilized to create the GF-7 Building dataset, as these two images (with the same viewing angle) can be employed to produce high-resolution (i.e., 0.65 m) and multi-bands (i.e., four bands) imagery.

**Table 2:** The parameters of the GF-7 DLC imagery.

GF-7 DLC imagery	Spectral range (µm)	Spatial resolution (m)	Viewing angles (°)	Width (km)
Multispectral image	Blue: 0.45–0.52	2.6	−5	≥20
	Green: 0.52–0.59			
	Red: 0.63–0.69			
	NIR: 0.77–0.89			
Panchromatic image	0.45–0.9	Forward: 0.8	Forward: +26	
		Backward: 0.65	Backward: −5	

The GF-7 Building dataset comprises ground-truth building labels along with four-bands multispectral GF-7 images, including RGB and NIR channels, with a resolution of 0.65 m. These GF-7 image tiles were collected from six GF-7 images, which captured both urban and rural regions in Tianjin, Lanzhou, Chongqing, Ningbo, Guangzhou, and Shenzhen (Table 3). Furthermore, these areas represent the diverse architectural landscapes of China’s inland and coastal regions. To create the 0.65-m GF-7 images, the 2.6-m GF-7 backward multispectral images and the 0.65-m backward panchromatic images were fused using the Gram-Schmidt algorithm^23^. Subsequently, all GF-7 images were projected into the universal transverse Mercator (UTM) projection with the WGS 84 datum and then stretched to the range of [0, 255] using the linear stretch algorithm.

**Table 3:** The file name of the selected GF-7 images.

City	File name
Tianjin	GF7_DLC_E117.2_N39.2_20201214_L1A0000272065
Lanzhou	GF7_DLC_E103.9_N36.0_20200331_L1A0000073869
Chongqing	GF7_DLC_E106.5_N29.7_20211023_L1A0000600549
Ningbo	GF7_DLC_E121.5_N29.9_20200210_L1A0000051030
Guangzhou	GF7_DLC_E113.3_N22.9_20200314_L1A0000064223
Shenzhen	GF7_DLC_E114.1_N22.5_20211114_L1A0000620133

### Dataset constructing

**Fig. 1:**
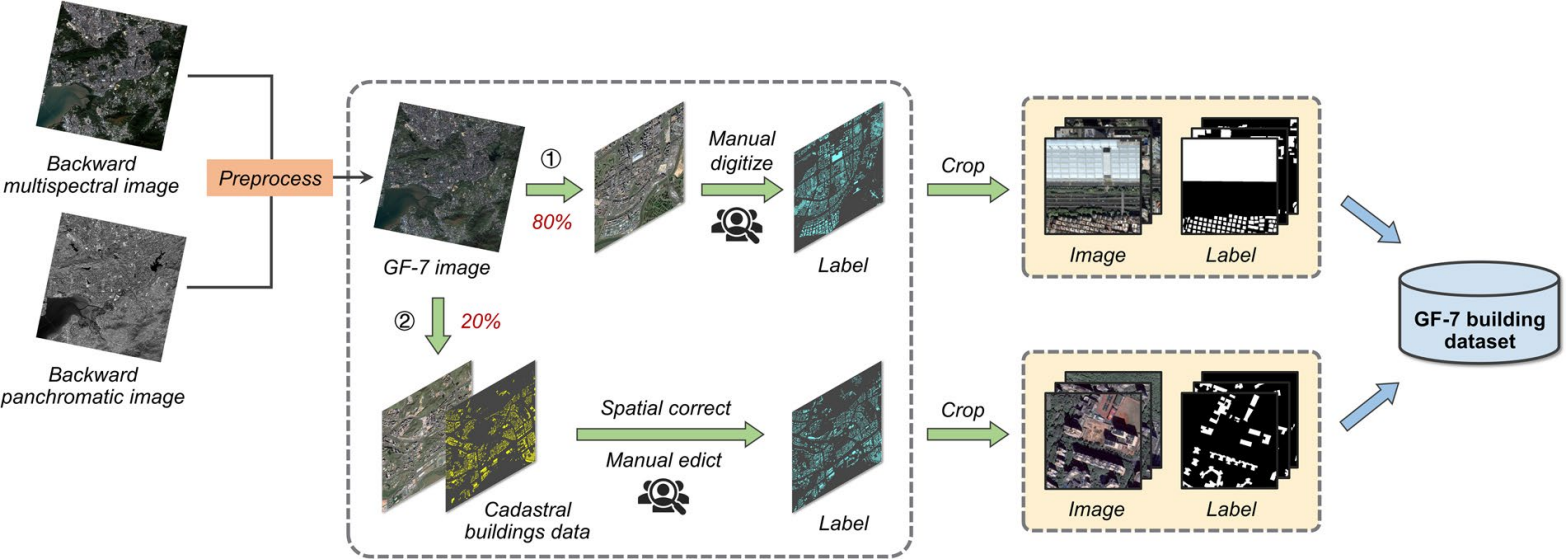
The framework for generating ground-truth building labels and the GF-7 Building dataset.

The general framework for generating ground-truth building labels is illustrated in Fig. 1. Approximately 80% of the building labels were created through manual digitization from GF-7 images, while the remaining 20% were generated by matching cadastral building data (i.e., building roof contour data) with the GF-7 images. Specifically, we primarily utilized cadastral building data as a reference and adjusted them to align with the GF-7 images using the spatial correction tool in ArcMap 10.7. Since there are still some obvious errors in the corrected cadastral buildings data, e.g., non-existing, missing, displaced, and inaccurate delineation, we further performed additional manual digitization and corrected the building roof contours using ArcMap 10.7. Finally, the accurate building vector maps were converted to raster images, where the building pixels and backgrounds are denoted as 255 and 0. To facilitate model training, both the GF-7 images and building labels maps were cut into nonoverlapping patches with a size of 512 × 512 pixels.

### Evaluation methods and training settings

Seven commonly used models for semantic segmentation were introduced to evaluate the performance of the GF-7 Building dataset. Specifically, the selected models were Fully Convolutional Network (FCN) 8S^24^, SegNet^25^, UNet^26^, RefineNet^27^, LinkNet^28^, Attention UNet^29^, and High-Resolution Network (HRNet)^30^. These models are typical CNNs proposed between 2015 and 2019. The FCN 8S leverages its dense prediction capabilities to effectively capture spatial hierarchies, while SegNet utilizes an encoder-decoder architecture to facilitate precise pixel-wise predictions. UNet employs a U-shaped topology, enabling high-resolution output by exploiting skip connections. The RefineNet architecture achieves refined segmentation by incorporating multi-path refinement modules. LinkNet prioritizes computational efficiency without compromising performance through its residual skip connections. The Attention UNet integrates attention mechanisms to selectively emphasize salient features during encoding and decoding. The HRNet preserves high-resolution representations by concurrently maintaining multi-scale information flow. Each of these models contributes distinct innovations to the field of deep learning-driven semantic segmentation, catering to diverse application scenarios and challenges.

As for model training, the input dimension of the model was 512 × 512 pixels for four bands (i.e., RGB and NIR), which were linearly stretched to the range of [0, 1]. The output of the model was patches of 512 × 512 pixels by one band, in which each pixel value denotes the probability of building a roof. All models were trained for 100 epochs with a batch size of 16. To optimize the training process, the binary cross-entropy (*L*_*BCE*_) loss function and Adam optimizer were adopted for our task^31^. Complementary to this, the initial learning rate was set at 0.001 and was halved when the loss value did not decrease over five epochs. All experiments were conducted using the Keras framework in Python 3.8 and executed on a computer equipped with an NVIDIA GeForce RTX 3090 Ti GPU.

1 $${L}_{BCE}=-\frac{1}{N}{\sum }_{i\,=\,1}^{N}[{y}_{i}\,\log (p({y}_{i}))+(1-{y}_{i})\log (1-p({y}_{i}))]$$

Where *y*_*i*_ is the reference building roof label for observation *i*, *p*(*y*_*i*_) is the predicted probability of building roof for observation *i*.

### Evaluation metrics

Quantitative evaluation metrics were calculated to assess the accuracy of the prediction, including Precision, Recall, F1-Score, overall accuracy (OA), intersection over union (IoU), and mean IoU (mIoU). Precision measures the accuracy for the building class and focuses on the correct positive predictions out of all positive predictions. Recall gives an offer for missing positive predictions. F1-Score offers the harmonic mean for both Precision and Recall. The overall accuracy, also referred to pixel accuracy, is used to evaluate segmentation performance. It is the ratio of the number of successfully predicted pixels to the total pixels in all patches of the test datasets. IoU measures the overlap between ground truth and prediction. mIoU is the mean of IoU^24^.

2 $$Precision=\frac{TP}{TP+FP}$$

3 $$Recall=\frac{TP}{TP+FN}$$

4 $$F1-Score=2\times \frac{Precision\times Recall}{Precision+Recall}$$

5 $$OA=\frac{TP+TN}{TP+TN+FP+FN}$$

6 $$IoU=\frac{TP}{TP+FN+FP}$$

7 $$mIoU=\frac{{\sum }_{i}^{n}IoU}{n}$$

Where TP, TN, FP, and FN denote true-positive, true-negative, false-positive, and false-negative, respectively.

## Data Records

### Repository and dataset format

**Fig. 2:**
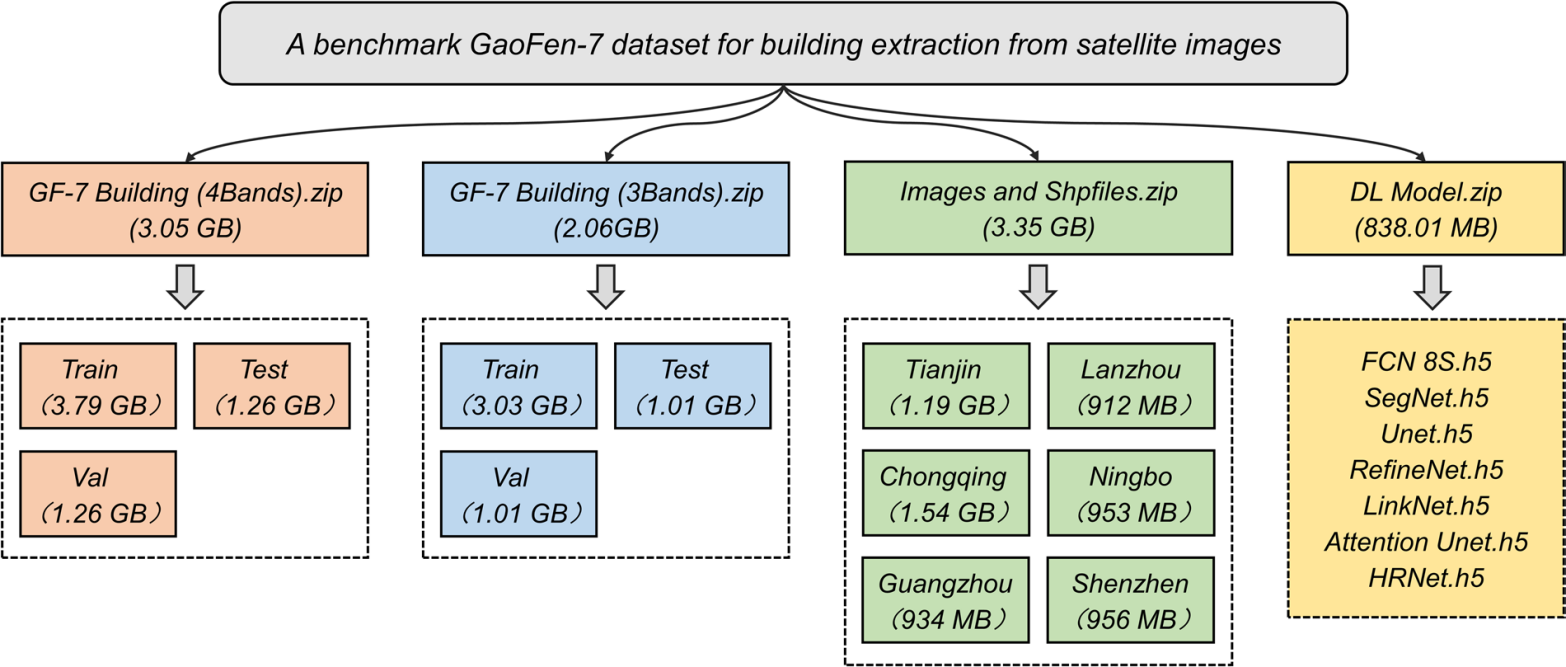
Data records for the GF-7 Building dataset.

The GF-7 Building dataset has been made public under Figshare (10.6084/m9.figshare.24305557)^32^. For the convenience of users, both the four-bands (RGB and NIR) and three-band (RGB) GF-7 Building datasets, which include the Train set.zip, Test set, and Validation set, have been shared. The datasets consist of images stored in TIFF format and adhere to a uniform naming convention of “city + number.” Furthermore, the complete building label Shpfiles and corresponding GF-7 images have been shared, which can be used to create building instance segmentation labels. In addition to the dataset, the well-trained DL models has been made publicly available in a zip archive named “DL Model” (Fig. 2).

### Dataset description

**Fig. 3:**
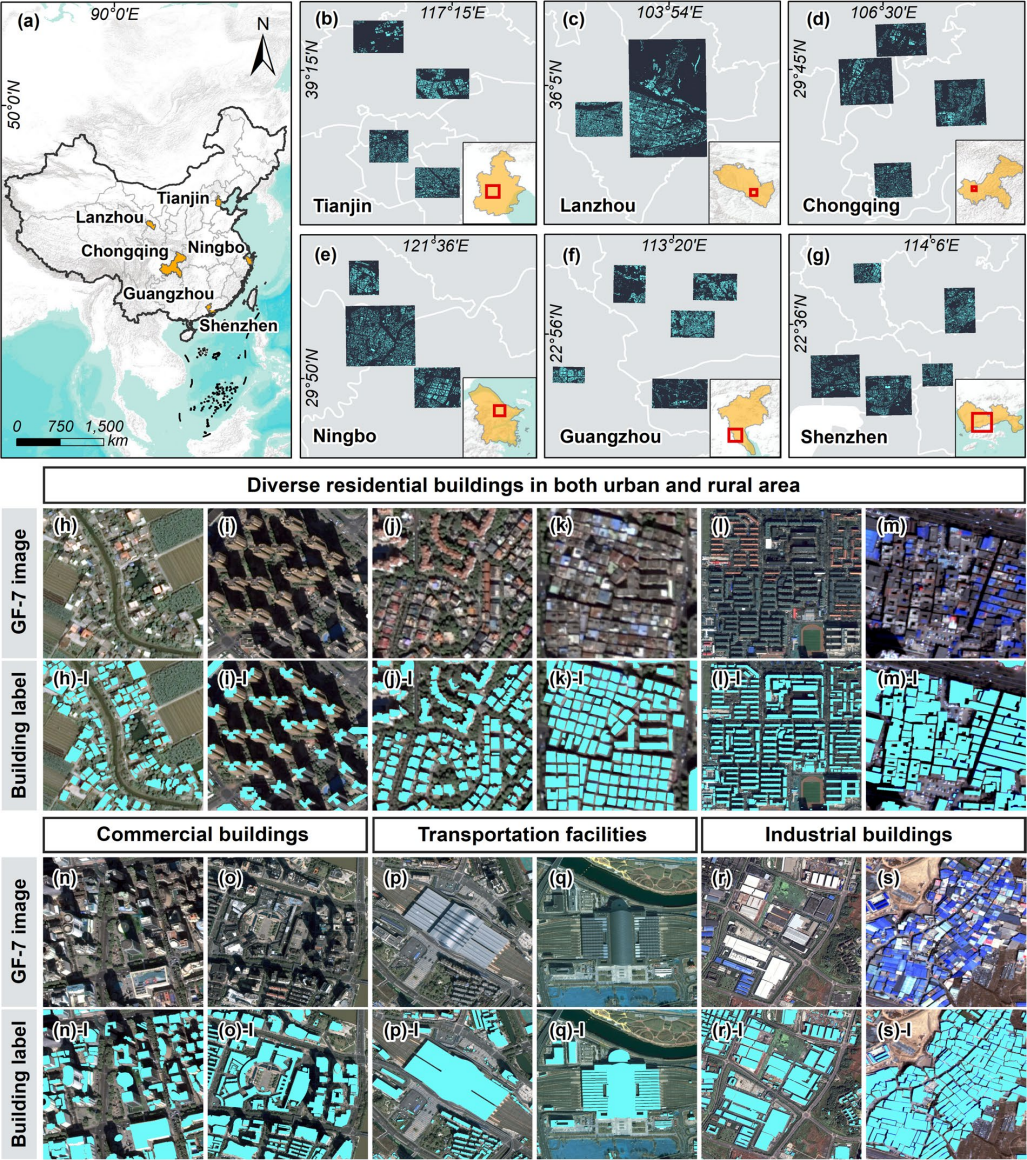
The geographic distribution of the GF-7 Building dataset and examples of various buildings in the GF-7 Building dataset.

The GF-7 Building dataset is constructed using six GF-7 images obtained in Tianjin, Lanzhou, Chongqing, Ningbo, Guangzhou, and Shenzhen (Fig. 3). These six cities are located in both coastal and inland areas of China and are typical Chinese cities with distinct built-up landscapes, city structures, and topographic features^33^. For example, the distribution of buildings in Tianjin, Lanzhou, Chongqing, and Ningbo is relatively regular. However, in Guangzhou and Shenzhen, the buildings are densely distributed and exhibit varying types and heights. These variations features make our dataset diverse and representative of China’s various built-up environments.

Unlike the existing building segmentation datasets mentioned in Table 1, the GF-7 Building dataset possesses the following noteworthy features:

#### Moderate-size Chinese satellite building segmentation dataset

**Fig. 4:**
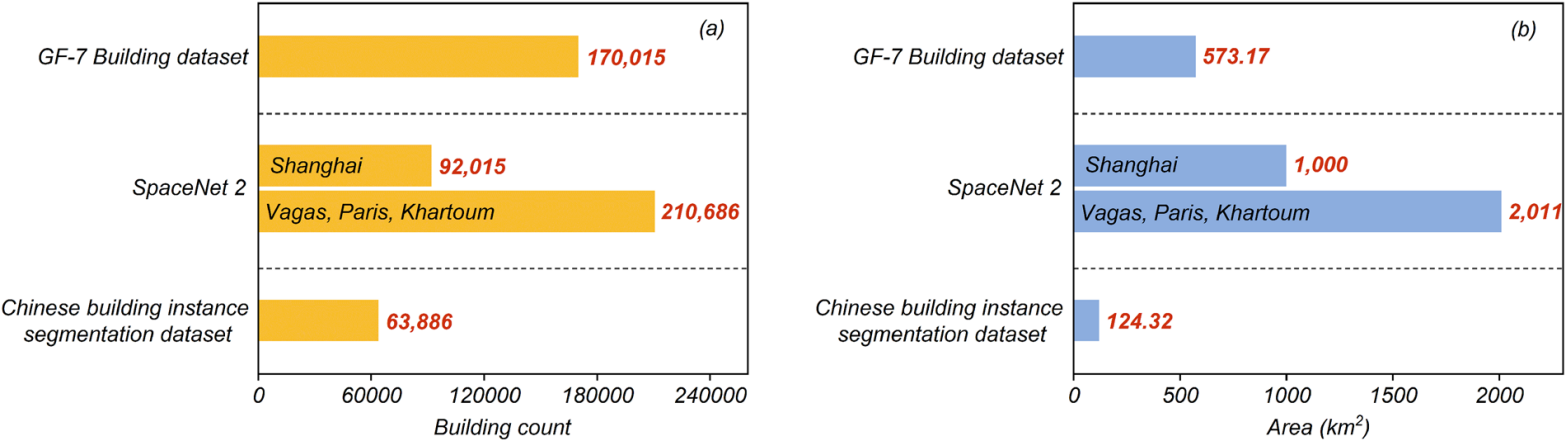
Comparison of the size of different building segmentation datasets. (**a**) Building count of the datasets. (**b**) The coverage area of the dataset.

We evaluated both the total number of annotations and the coverage area of our dataset as well as the CBISD^19^ and SpaceNet 2 dataset^16^. These datasets all cover localized areas of China to some extent. As exhibited in Fig. 4, CBISD contains 63,886 buildings, which is only about one-third of the total building count in our dataset. SpaceNet 2 features a total of 302,701 buildings, out of which 920,015 are located in China, accounting for about half the total building count in our dataset. Regarding the coverage area of these three datasets, our dataset’s coverage area is much smaller than that of SpaceNet 2. Nevertheless, the coverage area of our dataset is nearly five times larger than that of CBISD. In conclusion, our GF-7 building dataset is of moderate size and is more comprehensive than the building segmentation dataset within China.

#### High quality, high accuracy

**Fig. 5:**
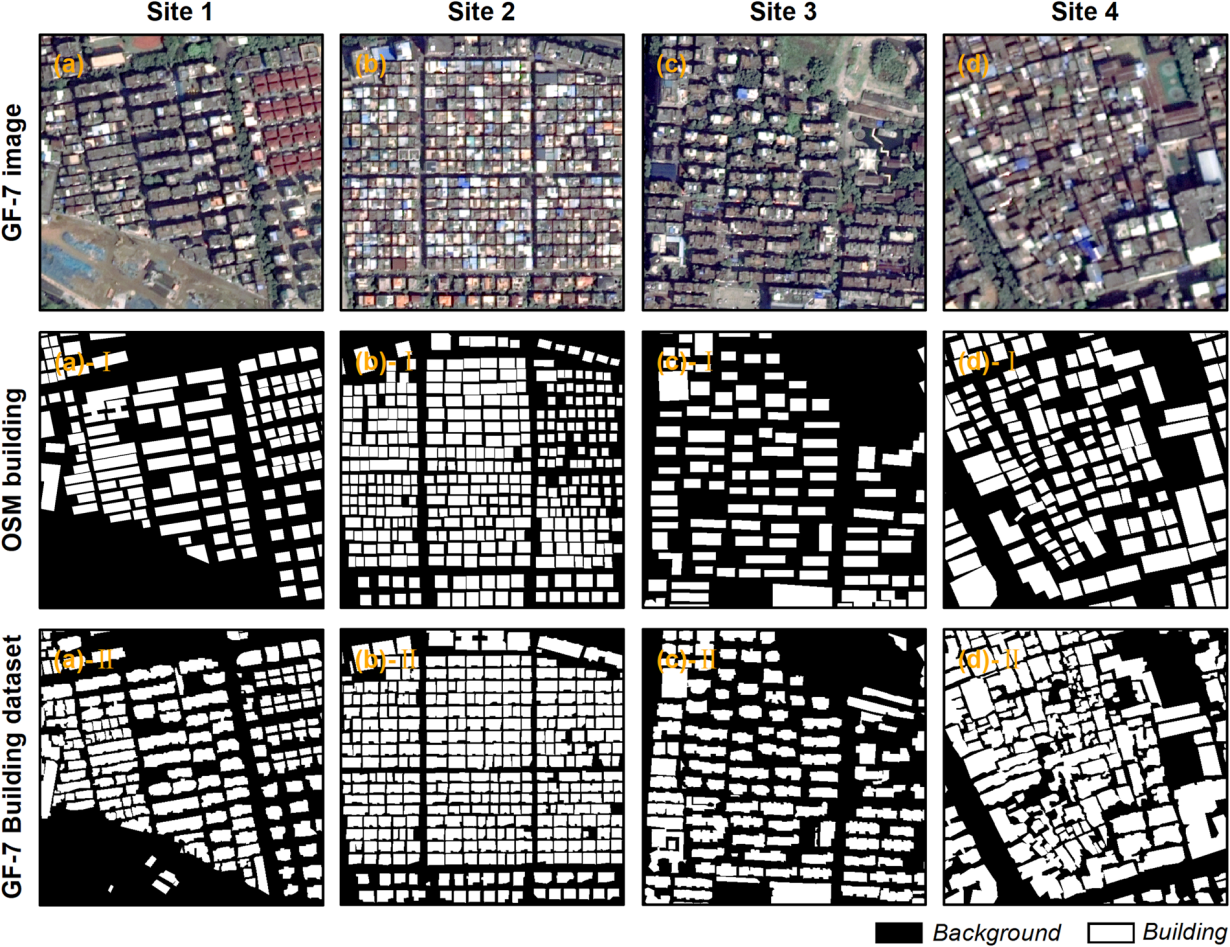
Examples of annotation details compared to the OSM building data.

The GF-7 Building dataset offers labels of exceptional quality and accuracy, primarily created through manual digitizing. Unlike simplified boundaries, such as the building data in OSM, the labels in the GF-7 Building dataset meticulously depict detailed contours of each building’s roofs (Fig. 5).

#### Multiple building sizes, types, heights, and multispectral

**Fig. 6:**
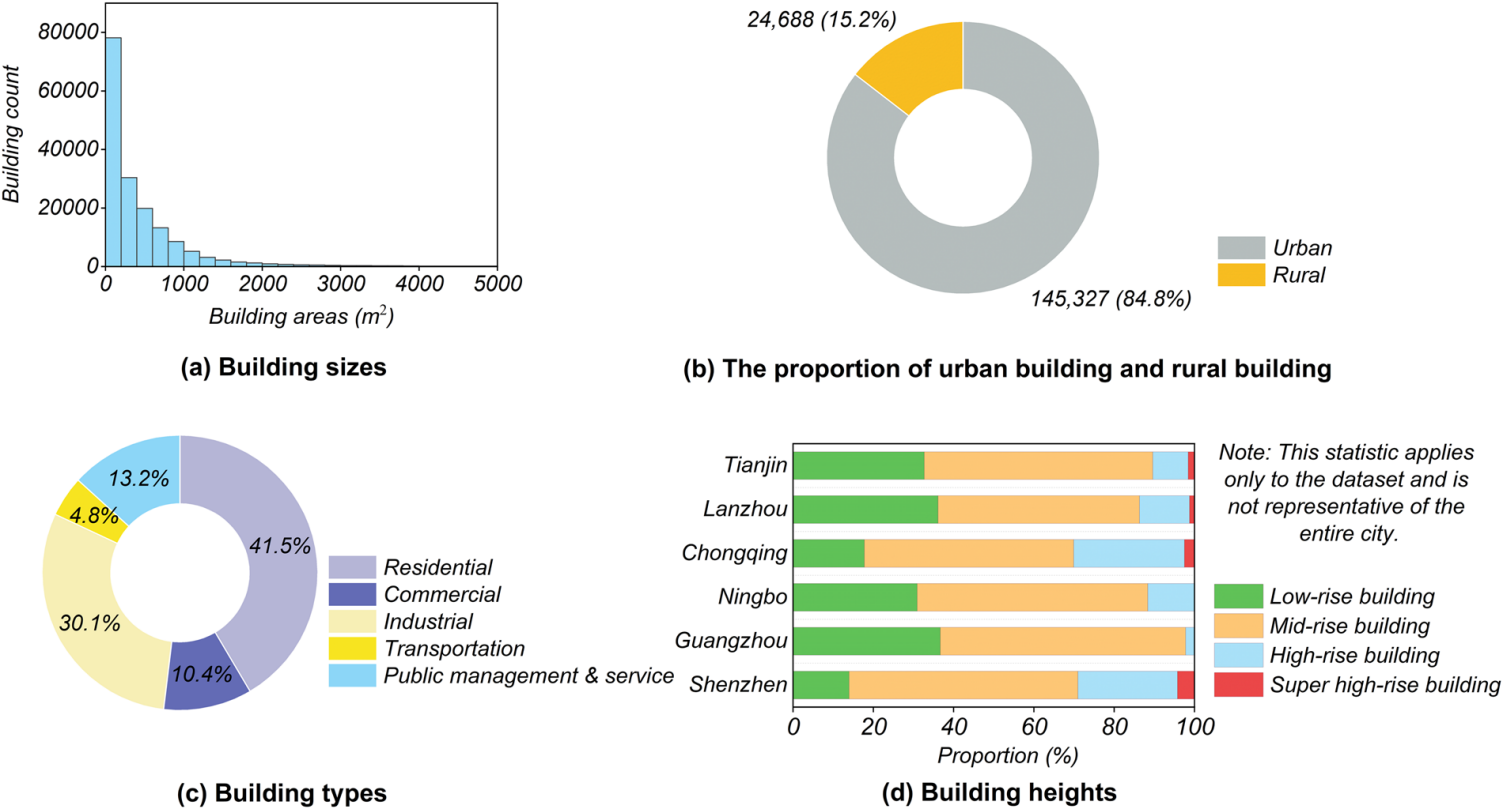
Statistics for the GF-7 Building dataset. (**a**) Distribution of building size. (**b**) The proportion of buildings in urban and rural areas. (**c**) The proportions of different types of buildings. (**d**) The proportion of buildings of different heights.

The building sizes in the GF-7 Building dataset span a range from 3 m^2^ to 1,480,000 m^2^ and are mainly concentrated within 1,000 m^2^ (Fig. 6a). Referring to the urban-rural distribution data released by the National Bureau of Statistics (http://www.stats.gov.cn/) in 2022, the GF-7 Building dataset comprises 24,688 (15.2%) rural buildings and 145,327 (84.8%) urban buildings (Fig. 6b). Among these, rural buildings are relatively singular, low-rise, and small, while urban buildings exhibit diversity in terms of size, structure, texture, height, and type. Additionally, rural buildings are characterized by low density and concentrated distribution, whereas urban buildings are featured by high density. Based on the EULUC-China data^34^, our dataset encompasses residential, commercial, industrial, transportation, and public management and service areas, with proportions of 41.50%, 10.39%, 30.10%, 4.78%, and 13.23%, respectively (Fig. 6c). Additionally, the dataset includes buildings of varying heights (Fig. 6d). According to the results of Chen *et al*.^33^, the distribution of building heights in the GF-7 Building dataset is as follows: low-rise buildings (i.e., below 9 m) account for 28.86%, mid-rise buildings (i.e., between 9 and 27 m) make up 56.66%, high-rise buildings (i.e., between 27 and 100 m) constitute 13.09%, and super high-rise buildings (i.e., taller than 100 m) represent 1.39% of the dataset. Furthermore, compared to other high-resolution satellite building datasets, the GF-7 Building dataset features “four-bands”, which provides richer spectral information and may contribute to improving the accuracy for building detection. The dataset’s diverse building types (i.e., various sizes, types, and heights) and its abundance of spectral and spatial information make it an ideal dataset for practical applications in building extraction, as well as for evaluating the potential of building extraction algorithms.

## Technical Validation

### Dataset splits

The GF-7 Building dataset consists of 5,175 pairs of 512 × 512 image tiles. Among them, there are 970, 722, 1,256, 745, 729, and 753 pairs of image tiles corresponding to Tianjin, Lanzhou, Chongqing, Ningbo, Guangzhou, and Shenzhen, respectively. To facilitate model training, the GF-7 Building dataset was divided into three distinct subsets: training, validation, and test sets, employing a split ratio of 6:2:2^35^. The dataset was not split randomly; rather, we carefully designed the splits to ensure that each set represents different types of buildings and is therefore representative of various scenarios. As a result, there are 3,106 pairs of image tiles for training, 1,034 pairs for validation, and 1,035 pairs for the test set (Table 4).

**Table 4:** Data division of the GF-7 Building dataset (Size: 512 × 512).

City	Training set (pairs)	Validation set (pairs)	Testing set (pairs)	Total		
				Tile count (pairs)	Building count	Area (km^2^)
Tianjin	582	194	194	970	23,576	107.43
Lanzhou	434	144	144	722	19,403	79.97
Chongqing	754	251	251	1,256	25,156	139.11
Ningbo	448	148	149	745	27,389	82.51
Guangzhou	437	146	146	729	46,484	80.74
Shenzhen	451	151	151	753	28,007	83.40
Total	3,106	1,034	1,035	5,175	170,015	573.17

### Performance among different DL models

**Fig. 7:**
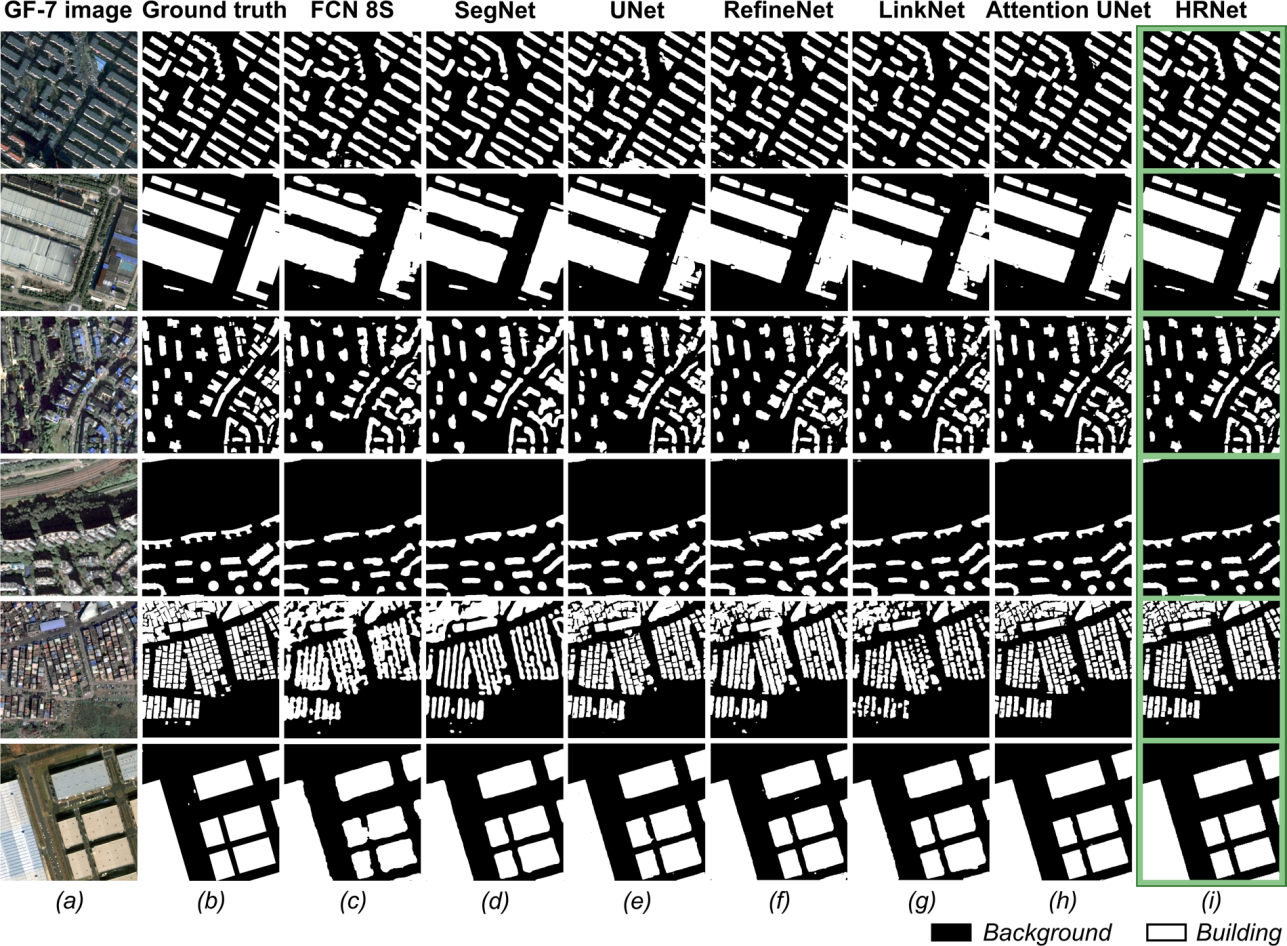
Semantic segmentation results of different DL methods on representative images from the GF-7 building test set. (**a**) GF-7 image. (**b**) Ground truth. (**c**–**i**) The results for different DL methods.

The performance of the GF-7 Building dataset was evaluated with seven DL methods. As shown in Table 5, regardless of the DL method, the OA on the test set is higher than 93%, and the Precision is above 82%. The HRNet, which is specially designed for preserving the information of small targets, surpasses other DL methods by leaps. Additionally, the Attention UNet shows considerable performance, with a 6.47% improvement in Precision compared to UNet. Figure 7 displays the building segmentation results of different DL methods on representative images from the GF-7 building test set. FCN 8S and SegNet are capable of predicting the area of the buildings, but they can hardly extract accurate edges of the buildings (Fig. 7c,d). In contrast, the other DL methods that utilize deep layers can achieve better performances (Fig. 7e–i). In particular, HRNet can better capture the details and boundary information of buildings due to its multi-scale fusion and high-resolution retention capabilities (Fig. 7i). Overall, the experiments demonstrate that the GF-7 Building dataset performs well and can be considered as a benchmark, and is useful for testing the extrapolation ability of DL methods.

**Table 5:** Semantic segmentation results obtained on the test set of the GF-7 Building dataset.

Method	Precision	Recall	F1-Score	OA	IoU	mIoU
FCN 8S	0.8213	0.7449	0.7812	0.9335	0.6410	0.7827
SegNet	0.8504	0.7945	0.8215	0.9449	0.6970	0.8170
UNet	0.8370	**0.8163**	0.8265	0.9454	0.7043	0.8208
RefineNet	0.8322	0.7459	0.7867	0.9355	0.6483	0.7876
LinkNet	0.8634	0.7216	0.7861	0.9374	0.6476	0.7884
Attention UNet	0.9017	0.7888	0.8415	0.9526	0.7264	0.8361
HRNet	**0.9118**	0.8132	**0.8597**	**0.9577**	**0.7539**	**0.8526**

### Performance for rural and urban building

To better illustrate the model’s performance in both rural and urban building, three typical rural building scenes were chosen for experiments^36^. These scenes are located in Foshan City, Guangzhou City, and Chongqing City, and include dense and sparse rural buildings. The size of each image is 1,024 × 1,024 pixels. Additionally, for comparison, three typical urban building scenes were chosen, encompassing high-rise commercial buildings and mid-rise residential buildings in the urban center of Shenzhen and Chongqing, along with industrial buildings in the suburban areas of Tianjin. The size of each urban image is 1,536 × 1,536 pixels. These six test areas, covering a total area of 8.41 km^2^, are located in different regions from the GF-7 Building dataset. To evaluate the model’s performance for rural and urban building, we conducted experiments with seven DL methods as mentioned above. The ground truth building roof maps were generated by manual digitization from GF-7 images using ArcGIS 10.7 for accuracy assessment.

**Fig. 8:**
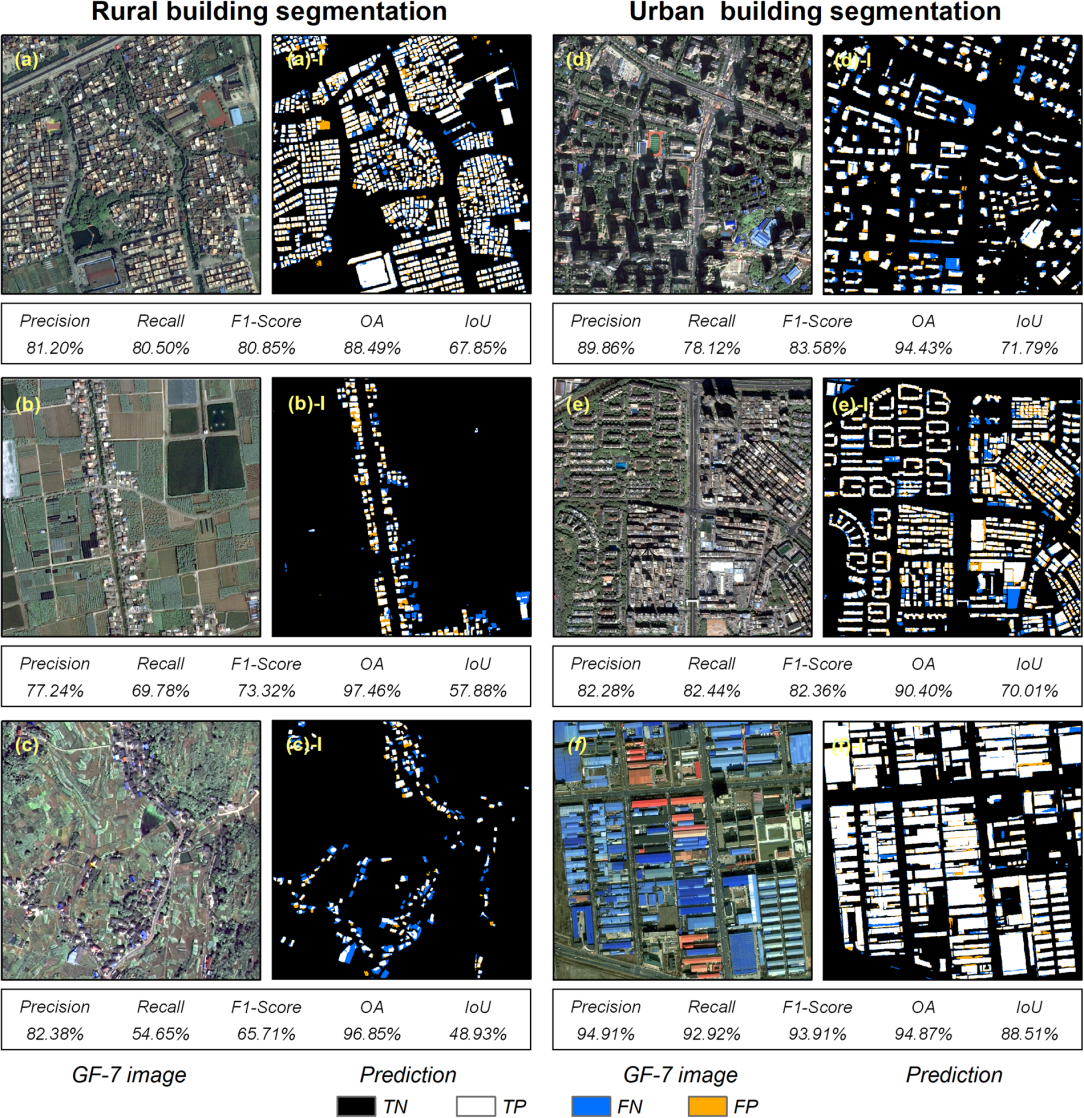
Performance of HRNet in rural and urban building detection. The abbreviations TN, TP, FN, and FP denote true-negatives, true-positives, false-negatives, and false-positives, respectively.

Taking HRNet as an example, it has achieved promising results in the detection of both rural and urban building, with OA ranging from 88.49% to 97.46% (Fig. 8). This demonstrates the usability of the GF-7 Building dataset for both rural and urban building extraction. Comparing the six test regions, it can be seen that the detection of urban buildings is significantly better than that of rural buildings, especially for the industrial buildings in the suburban area of the city, where the detection results are remarkable, with Precision, Recall, F1-Score, and OA all exceeding 90%. In addition, the Recall values for rural buildings range from 54.65% to 80.50%, while those for urban buildings range from 78.12% to 92.92%. The Recall values of rural buildings are significantly lower than those of urban buildings, indicating that the model has omissions in rural building detection. The experiments demonstrate that, combined with the classical DL methods, the GF-7 Building dataset can support the detection of different types of buildings in rural areas, urban centers and suburban areas. Omissions occur when the building and non-building categories are highly unbalanced (Fig. 8b–i, c–i).

**Fig. 9:**
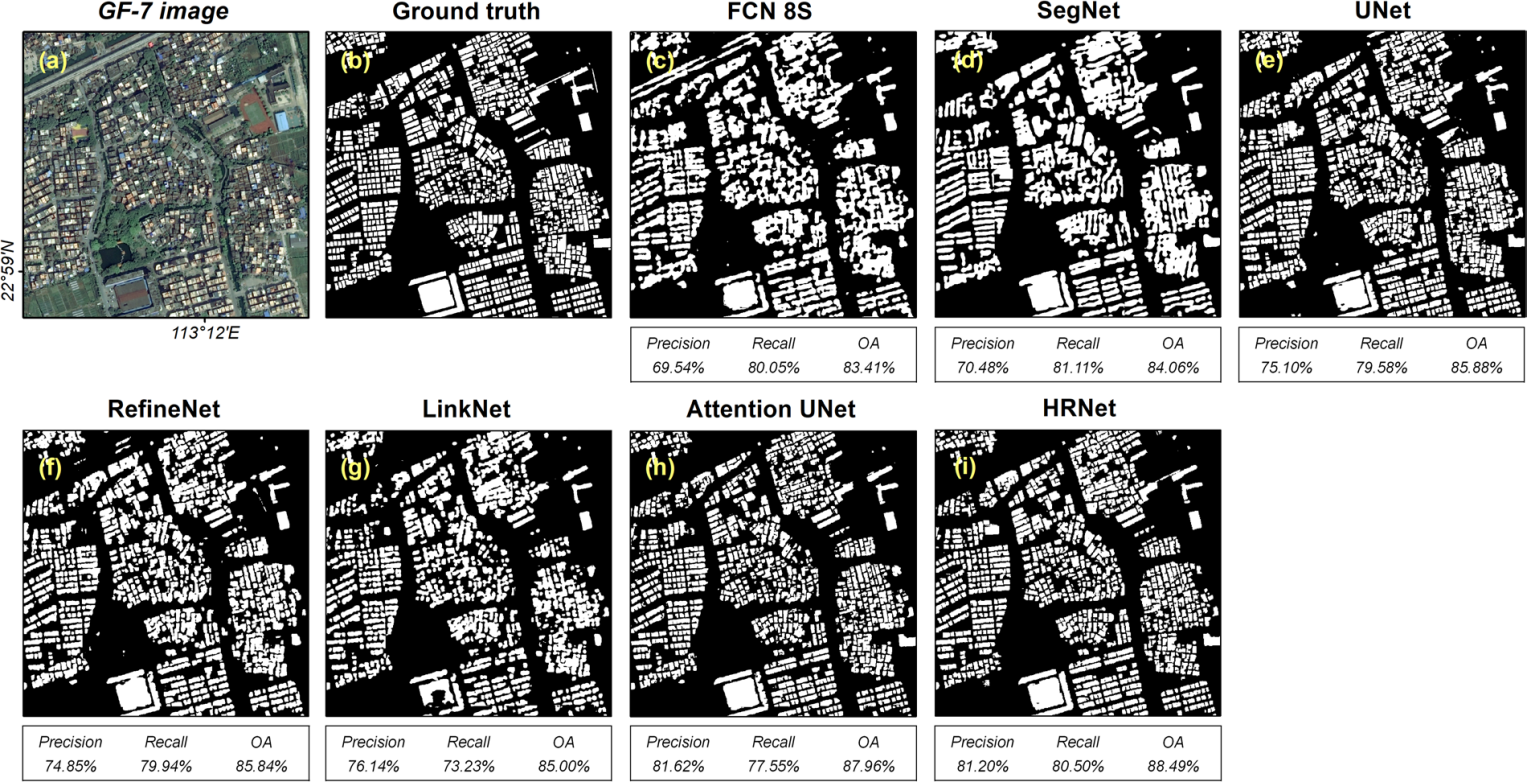
Rural building detection using classical DL methods based on the GF-7 Building dataset. (**a**) GF-7 image, (**b**) Ground truth building roof map, (**c**–**i**) Predictions by classical DL methods.

Furthermore, we conducted experiments using GF-7 imagery in the Xianyong village, which is located in Foshan city (Fig. 8a) to quantitatively compare the applicability of these classical DL methods for rural building detection. Based on the GF-7 Building dataset, classical DL methods are capable of detecting rural buildings, with OA ranging from 83.41% to 88.49%. However, there are variations in building detection details among different models (Fig. 9). Specifically, the segmentation results of FCN 8S and SegNet appear coarse, struggling to preserve the edge details of buildings. FCN 8S even misidentifies some roads as buildings. In contrast, UNet, RefineNet, LinkNet, Attention UNet, and HRNet exhibit better segmentation results, with HRNet performing optimally in detailing, effectively recognizing and distinguishing fine structures of rural buildings. Overall, the GF-7 Building dataset can essentially support rural building detection, but the specific detection performance is largely dependent on the structure of the model.

### Sample size and model performance

**Fig. 10:**
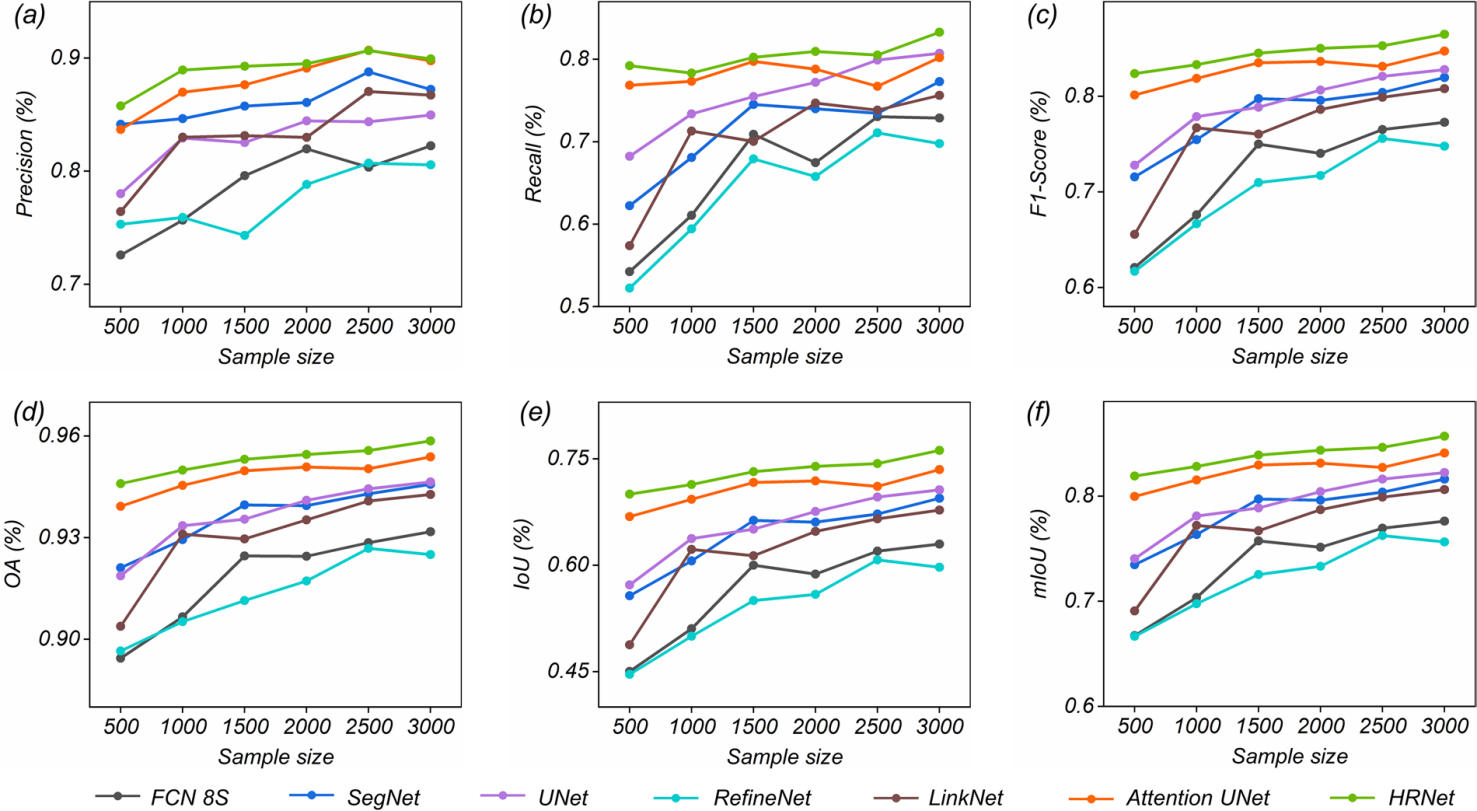
The relationship between sample size and model performance.

We divided the training samples and set the sample size to 500, 1,000, 1,500, 2,000, 2,500 and 3,000 to compare the performance of the DL methods under different sample sizes. As shown in Fig. 10, the prediction accuracy exhibited a rapid increase as the sample size expanded from 500 to 2,500 available images. When the sample size was increased from 2,500 to 3,000 images, a marginal improvement in prediction accuracy was observed. For instance, as the sample size expands from 500 to 2,500, the OA values for FCN 8S, SegNet, UNet, RefineNet, LinkNet, Attention UNet, and HRNet improve by 3.42%, 2.18%, 2.57%, 3.03%, 3.69%, 1.11%, and 0.98%, respectively. Additionally, when the sample size further increases from 2500 to 3000, the corresponding OA values change by 0.31%, 0.29%, 0.21%, −0.18%, 0.19%, 0.35%, and 0.28%. On the other hand, it can be observed that different DL methods exhibit inconsistent sensitivity to the sample size. For Attention UNet and HRNet, in the case of fewer samples (e.g., sample sizes less than 1,000), the models can still maintain relatively high prediction accuracy. The other five CNN models show the opposite trend—when there is an insufficient number of samples, the model’s prediction accuracy is comparatively low.

### Role of the NIR band

To explore the role of the additional NIR band of the GF7 Building dataset, comparison experiments were conducted to assess the prediction accuracy of DL methods under three bands (i.e., RGB) and four bands (i.e., RGB and NIR). All of the models were trained under the same conditions and parameters. The performance variation, obtained by subtracting the prediction accuracies of seven DL methods under the four bands (i.e., RGB and NIR) from those under the three bands (i.e., RGB), is presented in Table 6. Overall, the inclusion of the NIR band is generally beneficial for most methods. Specifically, most methods demonstrate positive effects, reflected in increased metrics such as Precision, Recall, F1-Score, OA, IoU, and mIoU, as indicated by positive bias values. This suggests that incorporating the NIR band contributes to improving the model’s detection performance for buildings. There are significant increases in Recall, with improvements of 2.12%, 3.10%, 6.01%, 10.46%, and 2.58% for FCN 8S, SegNet, UNet, RefineNet, and Attention UNet, respectively. The incorporation of the NIR band has demonstrated efficacy in mitigating omission detection for buildings. However, for some models, the addition of the NIR band did not lead to significant changes in accuracy, as exemplified by LinkNet. Therefore, in real-world applications, the decision to include the NIR band should be made by considering specific task requirements, data characteristics, and computational resources. Based on the conclusions of the above experiments, we release both the four-bands and three-bands versions of the GF-7 Building dataset for the convenience of users.

**Table 6:** Performance variation in building prediction with the addition of NIR band.

Method	Performance Variation (%)					
	Precision	Recall	F1-Score	OA	IoU	mIoU
FCN 8S	1.68	2.12	1.92	0.56	2.55	1.57
SegNet	−0.34	3.10	1.54	0.34	2.18	1.27
UNet	−3.11	6.01	1.82	0.26	2.60	1.42
RefineNet	0.49	10.46	6.41	1.40	8.27	4.85
LinkNet	0.41	−1.30	−0.60	−0.11	−0.82	−0.47
Attention UNet	−0.14	2.58	1.43	0.34	2.11	1.24
HRNet	0.26	0.04	0.14	0.05	0.21	0.13

### Transfer learning investigation

**Fig. 11:**
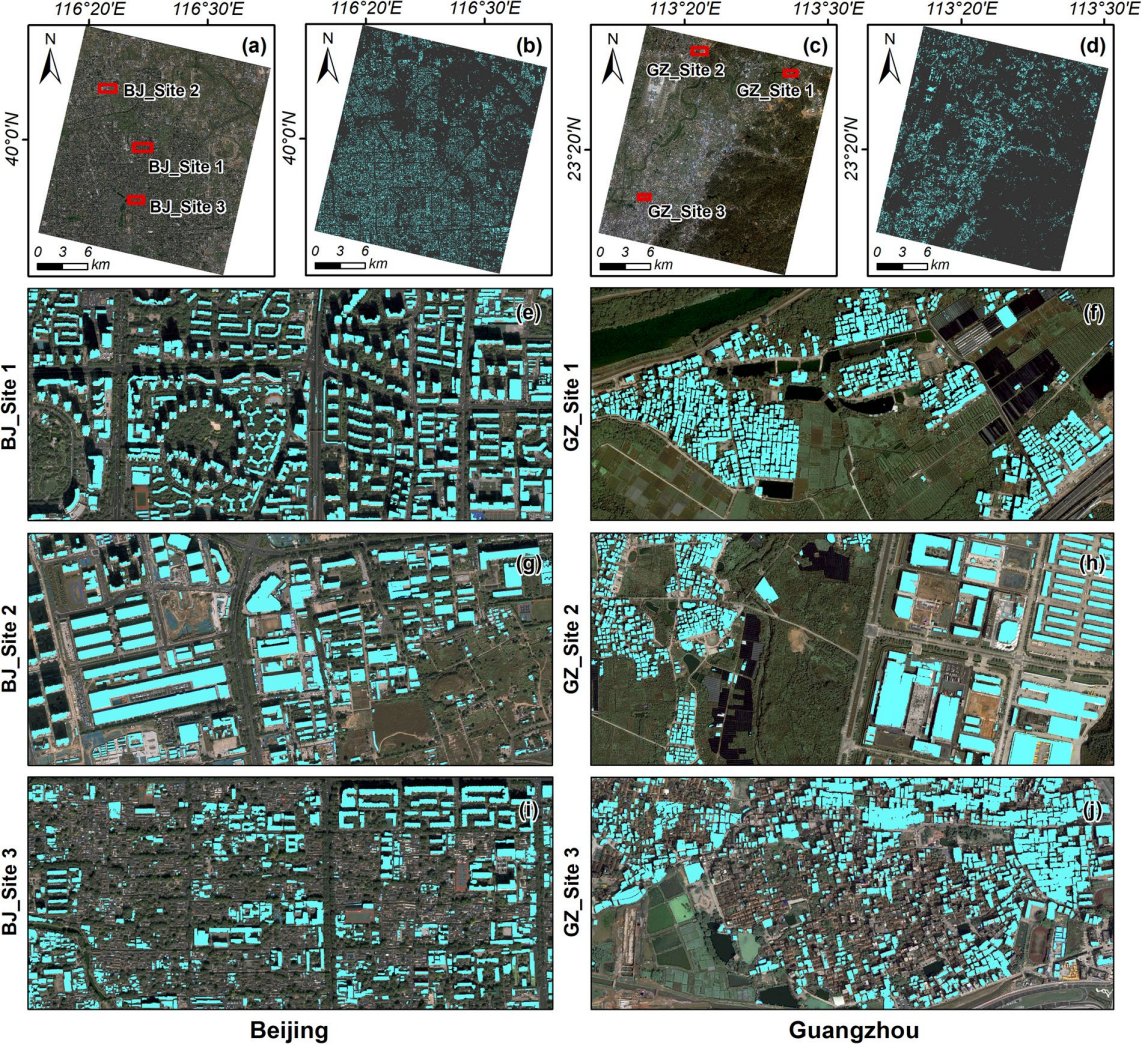
Direct prediction results of the well-trained HRNet model in Beijing and Guangzhou.

The above experiments were conducted on the GF-7 image tiles, leaving the transferability and practical application of the GF-7 Building dataset uncertain. In this section, we further explore the direct application of a well-trained DL model for building extraction from GF-7 satellite imagery over a large area. The well-trained HRNet was chosen for predicting buildings from the GF-7 images in Beijing and Guangzhou due to its proven superiority over other networks. Remarkably, these two GF-7 images cover cities from different regions in China, which have dramatically different types of landforms and buildings. The well-trained HRNet model was used to extract buildings from the processed GF-7 images of Beijing and Guangzhou. As shown in Fig. 11a–f, it can be observed that the well-trained HRNet model could effectively extract the buildings from the GF-7 images of Beijing and Guangzhou. This suggests that utilizing the well-trained model to predict buildings on GF-7 images in various regions could be a promising alternative. However, the well-trained HRNet model faced challenges in accurately identifying small and special buildings on the GF-7 images, such as small tile-roofed buildings (Fig. 11i–j). This limitation can be attributed to the lack of these special buildings in the GF-7 Building dataset and the limitation of image resolution. We are committed to expanding the GF7 building dataset in future research to encompass a wider range of building types.

## Usage Notes

The proposed GF-7 Building dataset has great potential for intelligent interpretation of buildings. It provides annotations for different types, shapes, sizes, spectra, and heights of buildings in China, and can serve as a building extraction benchmark for promoting the development of new algorithms for building detection. In the context of artificial intelligence, the GF-7 Building dataset can serve as a valuable data source for AI Big Models in the remote sensing field. Overall, considering the high quality, wide spatial distribution, and diverse building types, the GF-7 Building dataset represents a promising dataset for the intelligent interpretation of buildings. In our future work, we will keep expanding the GF-7 Building dataset in both size and type, making it more valuable for advancing research on building extraction from high-resolution satellite imagery. Moreover, we plan to convert our dataset into a building instance segmentation dataset to broaden its applications. We firmly believe that the GF-7 Building dataset will not only contribute to the development of new algorithms for building extraction but also facilitate large-scale intelligent interpretation of buildings in China.

## Data Availability

The programs and software used to generate all the results were Python and ESRI ArcMap 10.7. All DL models (FCN 8S, SegNet, UNet, RefineNet, LinkNet, Attention UNet, and HRNet) as well as the dataset are publicly available through the figshare repository (10.6084/m9.figshare.24305557)^32^.
